# Gut Microbiota-Targeted Photobiomodulation Ameliorates Alzheimer’s Pathology via the Gut–Brain Axis: Comparable Efficacy to Transcranial Irradiation

**DOI:** 10.3390/microorganisms13122659

**Published:** 2025-11-22

**Authors:** Shisheng Cao, Xinyu Shi, Yongqiang Chen, Tiaotiao Liu, Jiashen Hu, Xiaoxi Dong, Hongli Chen, Jianwu Dai, Huijuan Yin

**Affiliations:** 1 Integrative Regeneration Laboratory, Institute of Biomedical Engineering, Chinese Academy of Medical Sciences & Peking Union Medical College, Tianjin 300192, China; shengshi0730@163.com (S.C.); sxy19863825873@163.com (X.S.); chenyongqiang0830@163.com (Y.C.); hujiashen123@163.com (J.H.); chenhli0107@163.com (H.C.); jwdai@genetics.ac.cn (J.D.); 2 Tianjin Key Laboratory of Neuromodulation and Neurorepair, Tianjin 300192, China; 3 State Key Laboratory of Advanced Medical Materials and Devices, Tianjin 300192, China; 4 School of Biomedical Engineering and Technology, Tianjin Medical University, Tianjin 300070, China; liutt@tmu.edu.cn

**Keywords:** Alzheimer disease, light intervention, photobiomodulation, gut–brain axis

## Abstract

Alzheimer’s disease (AD) is a major neurodegenerative disorder with limited effective and affordable therapies. Photobiomodulation (PBM) offers a safe, non-invasive treatment strategy, yet conventional transcranial PBM (tc-PBM) is restricted by low skull penetration. To overcome this limitation, gut microbiota-targeted PBM (gm-PBM) has been proposed to modulate the gut–brain axis, though its efficacy and mechanisms remain unclear. Here, six-month-old APPswe/PS1dE9 mice received gm-PBM or tc-PBM (810 nm, 25 mW/cm^2^, 20 min/day for 4 weeks). Behavioral testing revealed that both treatments improved spatial learning and memory, while histological analyses showed reduced amyloid-β deposition and microglial shift toward an anti-inflammatory phenotype. Notably, gm-PBM specifically enriched short-chain fatty acid-producing bacteria, elevated propionate, butyrate, and secondary bile acids, and restored intestinal barrier integrity, whereas tc-PBM induced minimal microbiota changes. These findings suggest that gm-PBM confers neuroprotective effects comparable to or exceeding tc-PBM through modulation of the gut microbiota–metabolism–immune axis, highlighting its potential as a non-invasive and cost-effective therapeutic approach for AD.

## 1. Introduction

Alzheimer’s disease (AD) is one of the most common neurodegenerative disorders, with one new case of dementia occurring globally every three seconds [1]. In 2018, there were approximately 50 million AD patients worldwide, and this number is projected to rise to 152 million by 2050 [2]. The hallmark pathological features of AD include β-amyloid (Aβ) plaque deposition, abnormal Tau protein hyperphosphorylation, synaptic loss, and chronic neuroinflammation [3]. Although the underlying pathogenesis of AD remains incompletely understood, current pharmacological treatments (e.g., anti-Aβ monoclonal antibodies, secretase inhibitors) show limited efficacy and are costly, underscoring the urgent need to explore novel, safe, and effective therapeutic strategies [4,5,6,7].

In recent years, photobiomodulation (PBM) has garnered significant attention due to its high safety profile, non-invasiveness, and good tolerability [8]. Near-infrared (NIR) PBM can activate mitochondrial cytochrome c oxidase, enhance ATP production, and reduce reactive oxygen species (ROS) levels, thereby exerting neuroprotective effects [9,10,11]. Transcranial PBM (tc-PBM) has been shown to improve cerebral blood flow, suppress brain inflammation, and enhance gamma oscillations closely associated with cognitive function. However, the extremely low transmittance of light through the human skull (less than 5%) and the poor user experience of 40 Hz flickering light significantly limit the practicality of tc-PBM in home-based settings [12,13,14,15,16].

To overcome the limitations of light penetration, remote PBM strategies have emerged as a promising area of research [17]. Accumulating evidence suggests that AD is frequently accompanied by gut microbiota dysbiosis and impaired intestinal barrier function, characterized by reduced levels of short-chain fatty acids (SCFAs) and elevated lipopolysaccharides (LPS), the latter of which can activate microglia and exacerbate Aβ deposition [18,19]. Therefore, implementing gut microbiota-targeted photobiomodulation (gm-PBM) through modulation of the gut–brain axis may offer a novel therapeutic avenue for AD [20,21]. However, a systematic comparison between gm-PBM and tc-PBM remains lacking.

This study employed the APPswe/PS1dE9 mouse model and standardized PBM parameters (wavelength: 810 nm; power density: 25 mW/cm^2^; 20 min per day for 4 weeks) to systematically compare, for the first time, the therapeutic effects of gm-PBM versus tc-PBM on cognitive function and pathological features of AD. Furthermore, the study explored the potential mechanisms by which gm-PBM exerts its effects through modulation of the “gut microbiota–metabolism–immunity” axis. The results demonstrated that gm-PBM significantly improved learning and memory performance, effectively reduced cerebral Aβ plaque burden, restored intestinal barrier integrity, corrected SCFA and bile acid metabolic disturbances, and suppressed the pro-inflammatory phenotypes of microglia and macrophages. Notably, gm-PBM exhibited superior efficacy to tc-PBM in terms of metabolic reprogramming and inflammation resolution. These findings clarify the therapeutic advantages and underlying mechanisms of gm-PBM in AD, provide new experimental evidence supporting remote light-based modulation of the gut–brain axis, and highlight the promising potential of developing a safe, cost-effective, and home-compatible intervention strategy for AD management.

## 2. Materials and Methods

### 2.1. Animals and Animal Husbandry

All animal work was approved by the animal ethical and welfare committee at the Institute of Radiation Medicine, Chinese Academy of Medical Sciences & Peking Union Medical College (IRM-DWLL-2022239) and performed with strict adherence to the guidelines set out in the National Institutes of Health Guide for the Care and Use of Laboratory Animals. A total of 24 six-month-old female APPswe/PS1dE9 transgenic mice and 8 ten-month-old female wild-type (WT) C57BL/6 mice were purchased from Beijing HFK Bioscience Co., Ltd. (Beijing, China). Genotyping verification for the APPswe/PS1dE9 line was provided by the supplier. At approximately 6 months of age, APPswe/PS1dE9 mice typically exhibit prominent amyloid-β plaque deposition in the cortex and hippocampus, accompanied by mild microglial activation, early synaptic alterations, and subtle cognitive impairment. Mice were housed under a standard 12-h light/dark cycle, with four animals per cage, and were given free access to food and water. APPswe/PS1dE9 AD mice were randomly assigned to groups by the experimenter, with 8 mice in each group. Random numbers were generated using the standard = RAND () function in Microsoft Excel. Sample sizes were chosen in accordance with power analysis and with similar previously published experiments. Each mouse was used as one experimental unit.

### 2.2. LED Sources

The PBM devices used in this study were custom-developed in our laboratory for delivering tc-PBM and gm-PBM in APPswe/PS1dE9 mice. All LED modules were assembled and calibrated prior to experimentation.

A continuous-wave 810 nm LED was used as the light source. The output power was adjusted to achieve a stable irradiance of 25 mW/cm^2^ at the surface of the target region. Each mouse received irradiation for 20 min per day over a period of four weeks. The optical output was verified before each session to ensure consistent device performance.

For tc-PBM, the LED array was mounted onto a 3D-printed head-fixed helmet structure, designed using SolidWorks software (Version 2022, Dassault Systèmes, France) to ensure precise alignment over the prefrontal cortex (PFC) and hippocampal (HIPP) regions. The LED array was driven by a parallel–series hybrid circuit configuration, powered by an LT3465AES6 DC-DC constant-current driver. The output current was regulated using an external feedback resistor (R_FB). A passive heat sink was installed on the back of the LED board to prevent thermal buildup and to maintain the surface temperature below 37 °C during the irradiation period.

For gm-PBM, a 3D-printed arch-shaped mounting platform was used to hold a 4 × 3 LED array positioned above the abdominal region, enabling repeatable, stable, and non-contact illumination of the gut area. This configuration ensured consistent body positioning and irradiation alignment throughout the intervention.

### 2.3. Light Intervention

The mice were restrained and fixed in an awake state (with limbs taped), and the irradiation sites were treated with depilation cream. The exposed areas were then shaved and fully exposed before the LED device was placed on the abdomen (supine position) or the head (prone position) for irradiation. During the light exposure, the position of the light source was monitored to ensure that no displacement occurred.

The LEDs emitted light at a wavelength of 810 nm in continuous output mode, with a power density of 25 mW/cm^2^ and an irradiation time of 20 min.

AD mice in the sham group underwent the same procedures as the treatment groups, except that no light was applied, including the same duration of restraint. The control group consisted of healthy 10-month-old wild-type (WT) mice. To minimize subjective bias, cage labels indicating group names were replaced with serial number codes during the evaluation phase after the intervention.

### 2.4. Morris Water Maze

The Morris Water Maze (MWM) was used to assess cognitive ability in mice. After a 4-week course of light intervention, mice were rested for 1 week before MWM testing was started. First, mice were trained for 3 days to find a platform in the pool. This was followed by a 5-day learning ability assessment. The platform was placed in the first quadrant of the pool, and mice entered the water from each of the other three quadrants. Behavioral tracking and swim-path analysis were performed using the Tracking Master System, Version 4.0-MWM (Shanghai Fanbi Intelligent Technology Co., Ltd., Shanghai, China). A trial was considered successful when the mouse reached the hidden platform and remained on it for at least 3 s. The ≥3-s dwell criterion was adopted to distinguish intentional platform recognition from accidental contact, and this approach has been widely implemented in Morris water maze protocols to ensure that escape latency accurately reflects spatial learning performance. Escape latency was defined as the time from entry into the water to the moment the mouse continuously remained on the platform for ≥3 s. If a mouse failed to locate the platform within 120 s, a latency of 120 s was recorded, and the mouse was gently guided to the platform and allowed to remain there for 30 s. On the final day, the platform was removed and mice entered the water from any quadrant. The percentage of time spent in the platform area and the number of times the mice crossed the platform quadrant were calculated to assess the memory ability of the mice.

### 2.5. Reagents and Materials

All antibodies and reagents used in this study were commercially sourced. Primary anti bodies included Iba1 (Abcam, Cambridge, UK; ab178846), β-Amyloid 1–42 (Abcam, Cambridge, UK; ab201061), phospho-Tau (Ser396) (Abcam, Cambridge, UK; ab109390), F4/80 (Abcam, Cambridge, UK; ab300421), synaptophysin (ABclonal, Wuhan, China; A6344), COX-2 (Cell Signaling Technology, Danvers, MA, USA; 12282T), and CD163, ZO-1, and occludin (Proteintech, Wuhan, China; 16646-1-AP, 21773-1-AP, and 27260-1-AP).

Secondary antibodies included Cy^TM^3-conjugated donkey anti-rabbit IgG (Jackson ImmunoResearch, West Grove, PA, USA; 711-165-152) and Alexa Fluor^®^ 488 donkey anti-rabbit IgG (Jackson ImmunoResearch, 711-545-152). HRP-polymer detection reagents (anti-rabbit and anti-mouse) were obtained from Fuzhou New Step Biotechnology (Fuzhou, China; KIT-5005 and KIT-5002). Tyramide signal amplification (TSA) fluorophores, including Cy5-tyramide, Cy3-tyramide, and fluorescein-tyramide, were obtained from AAT Bioquest (Sunnyvale, CA, USA; 11066, 11065, and 11062).

### 2.6. Immunohistochemistry

Mouse tissue was prepared after MWM testing. Brain and colon tissues were rapidly removed and fixed for 24 h in 4% paraformaldehyde. The tissues were then dehydrated, embedded, and prepared as paraffin sections with a thickness of 5 μm. The tyramide signal amplification technique was used for multi-label immunofluorescence staining. Aβ1–42 and Iba1 were stained together to observe the pathological changes in AD plaques and brain inflammation. Co-staining of p-tau and synaptophysin was used to visualize nerve fibers and nerve axons. Aβ1–42, COX2, and CD163 were co-stained to observe the relationship between inflammation and plaques. For colon tissue, F4/80, ZO-1, and occludin were co-stained to observe intestinal inflammation and the intestinal barrier. For all image quantification, ImageJ 1.54f (http://imagej.org/, accessed on 10 February 2025) was used. The positive area percentage, plaque counts, and cell counts were analyzed for all experiments. To quantify the fraction of microglia overlapping with Aβ, we used clolc2 in ImageJ to analyze colocalization and acquire the MCC scores.

### 2.7. Gut Microbiota Genomics

Stool samples from the colons of mice were collected (n = 5). 16S rRNA gene amplicon sequencing analysis was then used to analyze the classification and abundance of mouse gut microbiota. Bacterial V3/V4 hypervariable regions of the bacterial 16S rRNA gene were amplified using a polymerase chain reaction (PCR) system with the following primers: 341F (5′-CTACGGGNGGCWGCAG) and 805R (3′-GACTACHVGGGTATCTAATCC). The PCR products were detected using electrophoresis and purified using magnetic beads. After purification, the PCR products were used as a two-round PCR template. The DNA was then detected and purified again before being quantified using a Qubit (Invitrogen Qubit 4 Fluorometer, Thermo Fisher Scientific, Waltham, MA, USA). Finally, the samples were mixed according to the concentration of PCR products and sequenced on the MiSeq platform (Illumina, San Diego, CA, USA).

Qiime 2 was used to analyze the data, and DADA2 was called to denoise the raw data. The redundancy of the denoised sequence was removed to obtain the feature information of the amplicon sequence variants (ASV). Species annotation was performed for each ASV to obtain the corresponding species information and species-based abundance distribution. The relative abundance, alpha diversity, beta diversity, and differences between groups were analyzed using the ASV_table. Principal coordinates analysis, principal component analysis, non-metric multidimensional scaling, and other dimensionality reduction diagrams and sample clustering trees were used to display the bacteria. The differential bacteria were screened based on linear discriminant analysis effect size analysis and a Kruskal–Wallis test. Furthermore, PICRUSt2 was used to predict the function of the flora; the databases included MetaCyc, Enzyme Commission (EC) Kyoto Encyclopedia of Genes and Genomes (KEGG), and Cluster of Orthologous groups (COG) of proteins.

### 2.8. Statistical Analyses

All data with error bars are represented as the mean ± SD. For comparisons between two groups, an unpaired two-tailed Student’s *t*-test was applied. For comparisons among more than two groups, one-way ANOVA was performed. *p* < 0.05 was considered significant. The GraphPad Prism (Version 9.5, GraphPad Software, San Diego, CA, USA) was used for the statistical analysis of data and figure production. For image quantification, ImageJ version 1.54f (http://imagej.org/, accessed on 10 February 2025) was used.

## 3. Results

### 3.1. Gm-PBM and Tc-PBM Treatments Can Attenuate Cognitive Dysfunction in AD-like Rats

**Figure 1:**
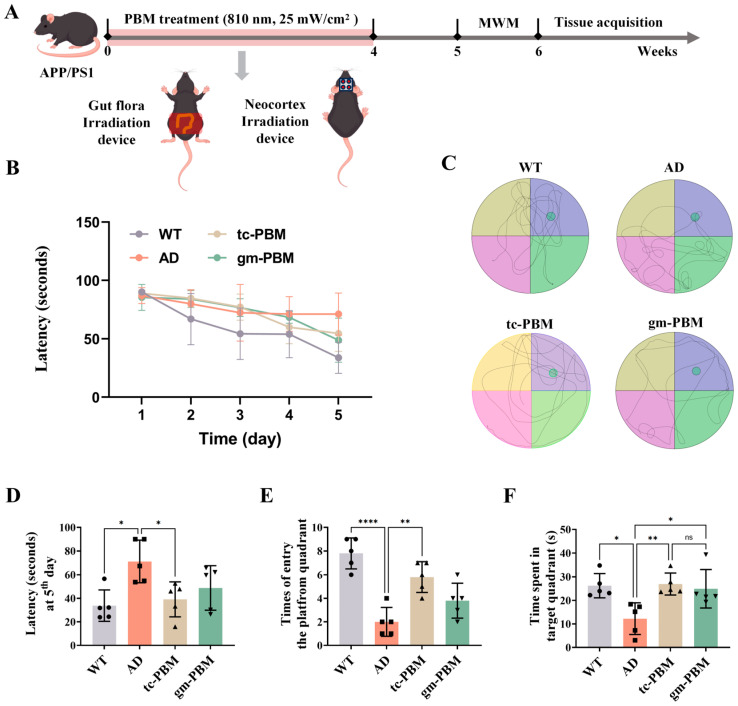
Gm-PBM and tc-PBM improve spatial learning and memory in APPswe/PS1dE9 mice. (**A**) Experimental timeline: APPswe/PS1dE9 mice received daily 810 nm PBM (25 mW/cm^2^, 20 min/day) for 4 weeks, applied either transcranially (tc-PBM) or to the abdominal gut (gm-PBM). In week 5, MWM testing was performed; tissues were collected in week 6. (**B**) Escape latency curves for each group (n = 5 per group). (**C**) Representative swim paths on day 5; the green circle denotes the former platform location. (**D**) escape latency on day 5, (**E**) number of entries into the target quadrant, and (**F**) time spent in the target quadrant. Data are mean ± SD (n = 5); one-way ANOVA with Tukey’s post hoc test, * *p* < 0.05, ** *p* < 0.01, **** *p* < 0.0001, ns, not significant.

To evaluate the effects of gm-PBM and tc-PBM on cognitive functions in APPswe/PS1dE9 mice, we administered daily light irradiation (wavelength: 810 nm; power density: 25 mW/cm^2^; duration: 20 min/day) to 6-month-old APP/PS1 mice for 4 consecutive weeks, followed by Morris water maze (MWM) testing during the 5th week (Figure 1A).

During the 5-day spatial acquisition training phase (Figure 1B–D), escape latencies among all groups did not significantly differ on day 2 (WT: 66.88 ± 21.96 s; AD: 79.96 ± 11.85 s). However, by day 5, the escape latency in AD mice significantly increased to 71.21 ± 17.99 s compared to WT controls (33.78 ± 13.34 s). gm-PBM treatment moderately reduced the latency to 48.80 ± 18.91 s, although this difference did not reach statistical significance when compared to the untreated AD group (*p* = 0.17). In contrast, tc-PBM intervention significantly shortened the latency further to 39.12 ± 14.85 s.

In the subsequent spatial probe test (Figure 1E,F), AD mice exhibited a significantly reduced number of entries into the target quadrant (2.0 ± 1.22 times) compared to WT mice (7.8 ± 1.3 times, *p* < 0.01). The gm-PBM treatment group showed a clear trend toward improvement in entry frequency (3.8 ± 1.48 times); although the difference did not reach statistical significance versus the AD group (*p* = 0.18), the numerical increase may hold biological relevance. Regarding time spent in the target quadrant, gm-PBM significantly prolonged the duration (24.92 ± 8.15 s) compared to the AD group (12.23 ± 6.72 s, *p* < 0.05), suggesting a beneficial effect on spatial memory. In comparison, tc-PBM treatment significantly increased both the number of entries (5.8 ± 1.48 times) and the duration spent (26.94 ± 4.67 s) in the target quadrant, demonstrating a more robust cognitive improvement in this behavioral paradigm.

### 3.2. Gm-PBM Reduced Aβ Production in the PFC and Hippocampus, Similar to Tc-PBM

To evaluate the therapeutic efficacy of gm-PBM and tc-PBM in alleviating AD pathology, we first examined whether these two light-based interventions could reduce β-amyloid (Aβ) plaque burden in the hippocampus (HIPP) and prefrontal cortex (PFC) of APP/PS1 transgenic AD mice.

**Figure 2:**
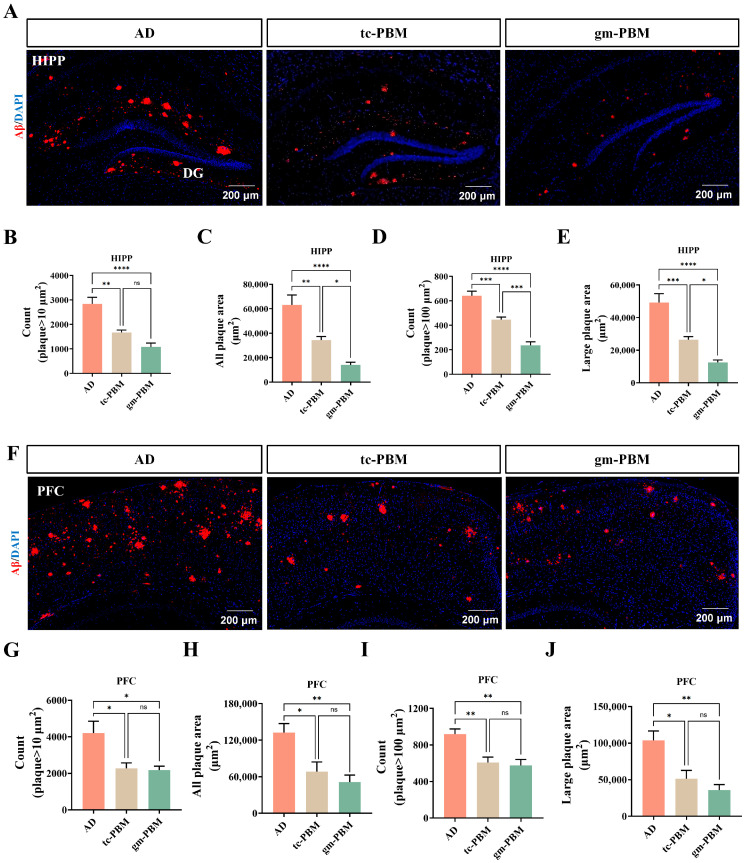
Gm-PBM and tc-PBM effectively clear Aβ plaques in APPswe/PS1dE9 mice. (**A**,**F**) Representative immunofluorescence images of β-amyloid (Aβ, red) counterstained with DAPI (blue) in the hippocampus (HIPP), including the dentate gyrus (DG; (**A**)). and prefrontal cortex (PFC; (**F**)). Shown are untreated AD, tc-PBM, and gm-PBM groups. (**B**–**E**) Quantification of hippocampal plaques: (**B**) number of plaques >10 μm^2^; (**C**) total plaque area; (**D**) number of large plaques >100 μm^2^; (**E**) cumulative area of large plaques. (**G**–**J**) Quantification of PFC plaques: (**G**) number of plaques >10 μm^2^; (**H**) total plaque area; (**I**) number of large plaques >100 μm^2^; (**J**) cumulative area of large plaques. mean ± SD, n = 6 per group, Statistics: one-way ANOVA followed by Tukey’s post hoc multiple-comparison test; * *p* < 0.05, ** *p* < 0.01, *** *p* < 0.001, **** *p* < 0.0001, ns = not significant.

Our results revealed that photobiomodulation exerted a robust effect in attenuating Aβ deposition. As shown in Figure 2A,F, the AD group exhibited dense red fluorescent Aβ plaques in both the hippocampus and PFC, while both gm-PBM and tc-PBM treatments markedly reduced plaque density. Quantitative analysis confirmed that both PBM modalities significantly decreased plaque burden, albeit to varying extents (Figure 2B–E). Specifically, in the hippocampus, gm-PBM and tc-PBM reduced total plaque number by 46% and 67%, and total plaque area by 42% and 61%, respectively, compared to the AD group. Notably, both treatments also significantly diminished the number and area of large plaques (>100 μm^2^).

Similar trends were observed in the PFC (Figure 2G–J). Overall, both gm-PBM and tc-PBM effectively reduced Aβ plaque accumulation in brain regions critical for cognition. However, gm-PBM exhibited superior clearance efficiency in several quantitative parameters, suggesting that remote gm-PBM may offer enhanced therapeutic benefits compared to direct transcranial irradiation.

### 3.3. Gm-PBM and Tc-PBM Increased the Synaptic Density

Neuronal apoptosis and synaptic loss are two additional key pathological features of AD. To determine whether different PBM strategies promote neuronal survival and synaptic restoration, we further examined their effects in relevant brain regions.

**Figure 3:**
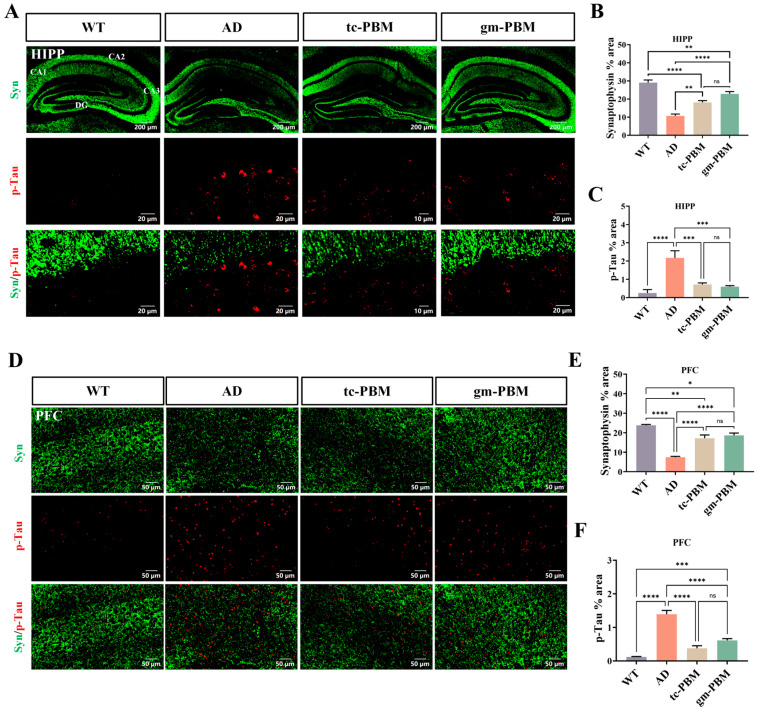
Gm-PBM and tc-PBM restore synaptic loss and abnormal Tau phosphorylation in APPswe/PS1dE9 mice. (**A**) Representative immunofluorescence images of the hippocampus showing amyloid plaque deposition across specific subregions, including CA1, CA2, CA3, and DG. Top row: synaptophysin (Syn, green) at low magnification (scale bar: 200 μm); middle row: phosphorylated Tau (p-Tau, red) at higher magnification (scale bar: 20 μm); bottom row: merged Syn/p-Tau images (scale bar: 20 μm). (**B**,**C**) Quantification of hippocampal regions: (**B**) Percentage of Syn-positive area; (**C**) Percentage of p-Tau-positive area. (**D**) Representative immunofluorescence images of the PFC, with layout and scale bars consistent with the hippocampal panel (overview: 50 μm; magnified views: 50 μm). (**E**,**F**) Quantification of PFC regions: (**E**) Percentage of Syn-positive area; (**F**) Percentage of p-Tau-positive area. mean ± SD, n = 6 per group, Statistical analysis: one-way ANOVA followed by Tukey’s post hoc multiple comparisons; * *p* < 0.05, ** *p* < 0.01, *** *p* < 0.001, **** *p* < 0.0001, ns = not significant.

In the hippocampus (Figure 3A–C), WT mice showed dense and continuous synaptophysin (Syn, green; a presynaptic marker) labeling, whereas p-Tau (red) was nearly undetectable. In contrast, APPswe/PS1dE9 mice exhibited a marked reduction in Syn signal, along with a mild increase in p-Tau labeling. It is important to note that this model does not typically develop classic neurofibrillary tangles; however, previous studies have reported age-dependent, Aβ-associated early elevations in p-Tau at this stage, which are interpreted as indicators of neuronal stress and synaptic dysfunction rather than mature tau aggregation. Therefore, the measurement of p-Tau in this study serves primarily to evaluate downstream Aβ-related neurotoxic responses rather than canonical tauopathy.

Compared with untreated AD mice, both gm-PBM and tc-PBM groups showed increased Syn expression accompanied by reduced p-Tau labeling, suggesting partial restoration of synaptic structure and cellular homeostasis. A similar tendency was observed in the prefrontal cortex (Figure 3D–F).

Collectively, both PBM strategies effectively restored synaptic marker expression and suppressed Tau hyperphosphorylation. Notably, gm-PBM demonstrated superior efficacy in repairing synaptic integrity specifically within the hippocampus, a brain region critical for memory formation, compared to its effects in the prefrontal cortex.

### 3.4. Gm-PBM and Tc-PBM Reduce Microglial Activation and Aβ Colocalization

Neuroinflammation plays a dual role in the pathology of AD, contributing to amyloid clearance during early stages but exacerbating disease progression in later phases. To further elucidate the effects of PBM on AD-associated neuroinflammation, we examined the area fraction of ionized calcium-binding adapter molecule 1 (Iba1)-positive microglia in the PFC and hippocampus, as well as their spatial association with Aβ plaques.

**Figure 4:**
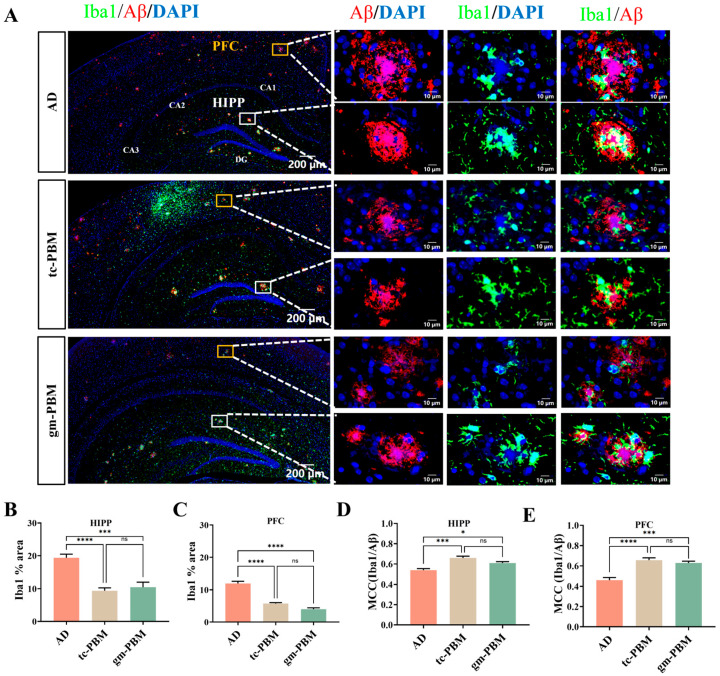
PBM treatment reduces microglial aggregation and Iba1/Aβ colocalization in the brains of APPswe/PS1dE9 mice. (**A**) Representative triple immunofluorescence images of the hippocampus and cortex showing Iba1 (microglia, green), Aβ (red), and DAPI (blue). Hippocampal panels include defined subregions: CA1, CA2, CA3, and the DG. Overview images (left; scale bar: 200 μm) and enlarged views of two representative plaques (right; scale bar: 10 μm) are shown for the AD, tc-PBM, and gm-PBM groups. Enlarged panels display Aβ/DAPI, Iba1/DAPI, and merged Iba1/Aβ staining. (**B**,**C**) Quantification of Iba1-positive area: (**B**) hippocampus; (**C**) prefrontal cortex (PFC). (**D**,**E**) Quantification of Mander’s colocalization coefficient: (**D**) colocalization of Iba1 and Aβ in the hippocampus; (**E**) colocalization of Iba1 and Aβ in the PFC. mean ± SD, n = 6 per group, Statistical analysis: one-way ANOVA followed by Tukey’s post hoc test; * *p* < 0.05, *** *p* < 0.001, **** *p* < 0.0001; ns indicates no significant difference.

As shown in Figure 4A–C, AD mice exhibited substantial microglial activation, with Iba1-positive areas reaching 19.38 ± 2.06% in the hippocampus and 11.96 ± 1.77% in the PFC. Both gm-PBM and tc-PBM significantly reduced Iba1 coverage in these regions. No significant differences were observed between the two PBM treatment groups, indicating that both PBM strategies effectively attenuated pathological microglial aggregation.

Mander’s colocalization coefficient (MCC) analysis further revealed that in AD mice, the Iba1/Aβ colocalization coefficients were 0.54 ± 0.04 in the hippocampus and 0.46 ± 0.07 in the PFC (Figure 4D,E). tc-PBM treatment increased these values to 0.66 ± 0.04 and 0.66 ± 0.05, respectively, while gm-PBM yielded coefficients of 0.61 ± 0.07 (HIPP) and 0.63 ± 0.04 (PFC). These results suggest that PBM enhances the spatial overlap between microglia and residual Aβ plaques, potentially reflecting elevated microglial phagocytic activity toward Aβ following intervention.

### 3.5. Comparable Immunomodulatory Effects of Tc-PBM and Gm-PBM

**Figure 5:**
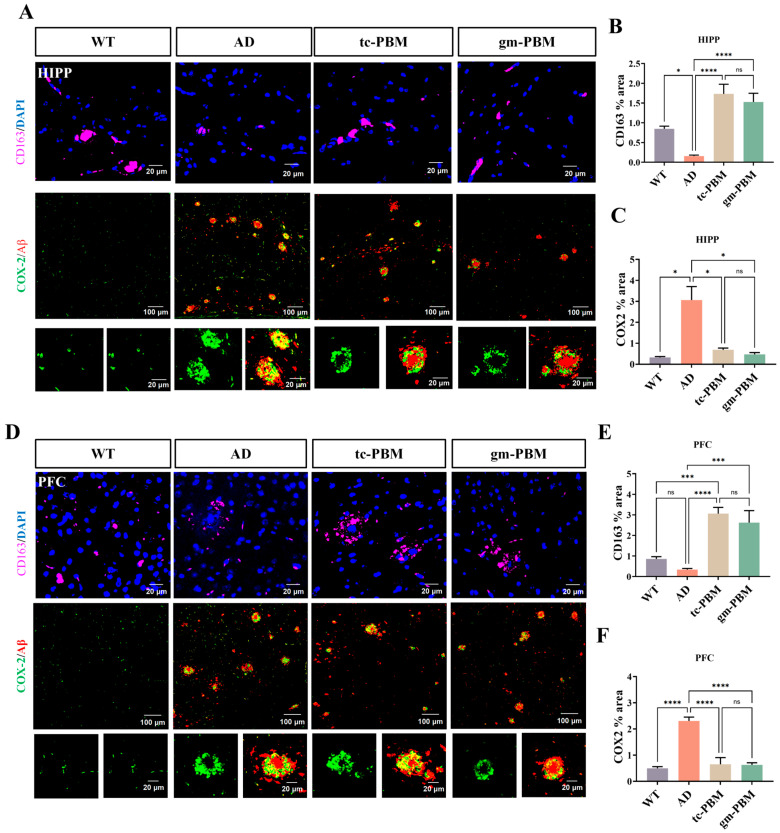
PBM treatment increases CD163 expression and suppresses COX-2 expression in the brains of APPswe/PS1dE9 mice. (**A**) Representative immunofluorescence images of the hippocampus showing CD163 (magenta) and DAPI (blue) in the upper row (scale bar: 20 μm), as well as dual staining of COX-2 (green) and Aβ (red) in the middle row (overview, scale bar: 100 μm) and bottom row (magnified views, scale bar: 20 μm). (**B**,**C**) Quantitative analysis of the hippocampus: (**B**) Percentage of CD163-positive area; (**C**) Percentage of COX-2-positive area. (**D**) Representative immunofluorescence images of the prefrontal cortex (PFC), showing CD163 and COX-2/Aβ staining as in (**A**) (overview: 100 μm; magnified: 20 μm). (**E**,**F**) Quantitative analysis in the PFC: (**E**) Percentage of CD163-positive area; (**F**) Percentage of COX-2-positive area. mean ± SD, n = 6 per group. Statistical analysis: one-way ANOVA followed by Tukey’s post hoc test; * *p* < 0.05, *** *p* < 0.001, **** *p* < 0.0001; ns indicates no significant difference.

The polarization of microglia toward either the M1 (pro-inflammatory) or M2 (anti-inflammatory) phenotype is influenced by pathological insults, inflammatory conditions, and therapeutic interventions. In the untreated AD group, the proportion of M2-type microglia—labeled by CD163—was significantly reduced in the hippocampus compared to the WT group, while the expression of cyclooxygenase-2 (COX-2), a marker of M1-type microglia, was markedly elevated. These findings indicate a phenotypic shift toward pro-inflammatory activation, characterized by a decrease in anti-inflammatory microglia and upregulation of inflammatory mediators (Figure 5A–C).

Following gm-PBM and tc-PBM treatments, the number of CD163-positive cells in the hippocampus increased significantly, accompanied by a notable reduction in COX-2 expression. Similar trends were observed in the PFC (Figure 5D–F).

These results suggest that PBM intervention effectively restores the M2 anti-inflammatory microglial phenotype (CD163) and suppresses the expression of the pro-inflammatory enzyme COX-2. Both PBM modalities demonstrated comparable efficacy, indicating that gm-PBM is as effective as tc-PBM in modulating neuroimmune responses in the AD brain.

### 3.6. Gm-PBM Confers Superior Protection on Gut Barrier Compared to Tc-PBM

In addition to its beneficial effects on the central nervous system, whether PBM can modulate peripheral gut homeostasis via the gut–brain axis represents a crucial aspect of its therapeutic potential. To address this, we further evaluated the impact of different PBM strategies on intestinal inflammation and gut barrier integrity. The results demonstrated that gm-PBM exhibited a notable advantage in alleviating intestinal inflammation in AD model mice.

**Figure 6:**
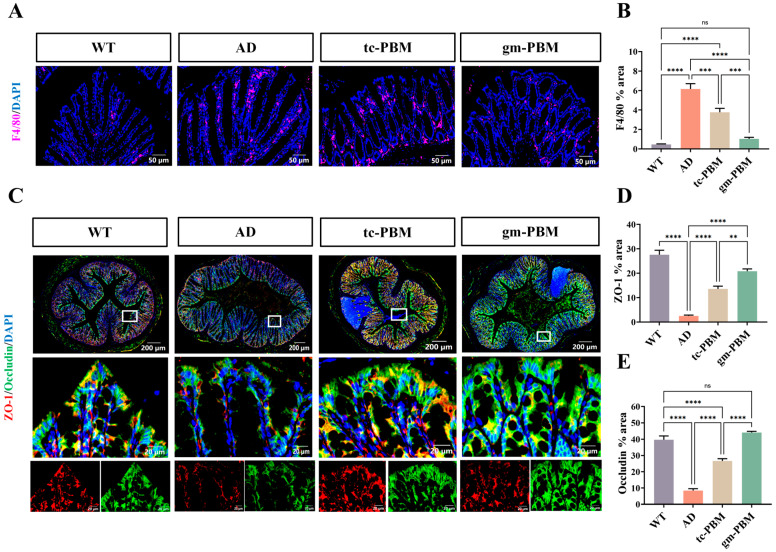
Gm-PBM intervention reduces intestinal macrophage infiltration and restores epithelial tight junction proteins in APPswe/PS1dE9 mice. (**A**) Representative immunofluorescence images of colonic tissue showing F4/80 (macrophage marker, red) and DAPI (nuclei, blue) staining. Scale bar: 50 μm. (**B**) Quantification of F4/80-positive area. (**C**) Representative immunofluorescence images of tight junction proteins in colonic tissue: ZO-1 (red), Occludin (green), and DAPI (blue), showing whole-section views (top row, scale bar: 200 μm) and enlarged views (bottom row, scale bar: 20 μm). (**D**,**E**) Quantitative analysis of tight junction proteins: (**D**) ZO-1-positive area (%); (**E**) Occludin-positive area (%). mean ± SD, n = 6 per group. Statistical analysis: one-way ANOVA with Tukey’s post hoc test; ** *p* < 0.01, *** *p* < 0.001, **** *p* < 0.0001; ns indicates no significant difference.

Using F4/80 as a macrophage marker, we observed a marked increase in F4/80-positive area in the colons of AD mice, indicating extensive infiltration of inflammatory cells (Figure 6A). tc-PBM treatment significantly reduced this area to 3.77 ± 1.1%, whereas gm-PBM achieved a further reduction to 1.03 ± 0.36%, suggesting that remote modulation via the gut–brain axis is more effective in suppressing localized intestinal inflammation (Figure 6B).

Moreover, tight junction proteins ZO-1 and Occludin were significantly disrupted in the AD group: the ZO-1-positive area decreased from 27.57 ± 5.04% in WT mice to 2.51 ± 0.89%, and Occludin from 39.72 ± 5.64% to 8.46 ± 2.8%. tc-PBM treatment partially restored ZO-1 (13.58 ± 2.63%) and Occludin (26.66 ± 3.42%) levels. In contrast, gm-PBM led to more robust recovery, increasing ZO-1 and Occludin expression to 20.83 ± 2.31% and 44.09 ± 1.54%, respectively (Figure 6C–E).

Collectively, these findings indicate that gm-PBM not only significantly mitigates AD-associated intestinal inflammation but also more effectively restores epithelial barrier integrity compared to tc-PBM, further supporting its therapeutic potential in modulating the gut–brain axis.

### 3.7. Gm-PBM Effectively Rectifies Gut Microbiota Dysbiosis in AD Model Mice

**Figure 7:**
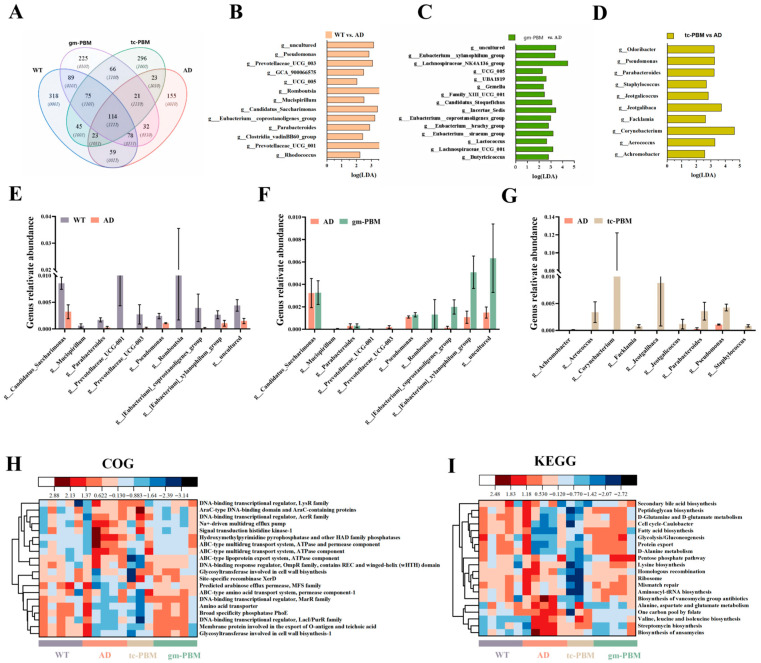
Gm-PBM intervention remodels gut microbiota composition and function in APPswe/PS1dE9 mice. (**A**) Venn diagram showing the shared and unique ASVs among four groups: WT, AD, tc-PBM, and gm-PBM C. (**B**–**D**) Differential microbial biomarkers identified by linear discriminant analysis (LDA, LDA score >3.0): (**B**) Comparison between WT and AD; (**C**) Comparison between gm-PBM and AD; (**D**) Comparison between tc-PBM and AD. (**E**–**G**) Bar plots showing the relative abundance of representative bacterial genera: (**E**) WT vs. AD; (**F**) AD vs. gm-PBM; (**G**) AD vs. tc-PBM. (**H**,**I**) (mean ± SD, n = 5/group) Functional prediction heatmaps illustrating changes in pathway abundance across groups based on COG (**H**) and KEGG (**I**) annotations (Z-score scaled).

Venn diagram analysis (Figure 7A) revealed 114 ASVs shared between the AD and WT groups, while gm-PBM and tc-PBM interventions selectively enriched 45 and 66 unique ASVs, respectively. Linear discriminant analysis (LDA) (Figure 7B–D) further indicated that, compared to WT mice, the AD group was enriched with potentially pathogenic genera such as *Parabacteroides*, *Pseudomonas*, and *Romboutsia*, while short-chain fatty acid SCFA-producing taxa including Butyricicoccus, Lachnospiraceae_NK4A136_group, and Eubacterium_siraeum_group were markedly reduced. gm-PBM intervention (Figure 7C) significantly enriched beneficial SCFA-producing genera such as *Butyricicoccus*, *Lactococcus*, and multiple members of the *Eubacterium* genus (LDA > 3.5). In contrast, tc-PBM (Figure 7D) primarily enriched genera like *Aerococcus*, *Corynebacterium*, and *Jeotgalicoccus*.

Bar plots further illustrated (Figure 7E) a significant reduction in SCFA-producing bacteria including Eubacterium_coprostanoligenes_group and *Butyricicoccus* in the AD group, accompanied by a pronounced increase in pathogenic *Pseudomonas*. gm-PBM treatment (Figure 7F) markedly restored the abundance of these SCFA-producing genera and significantly reduced Pseudomonas levels. tc-PBM (Figure 7G) also exerted partial restorative effects on these taxa, although to a lesser extent compared to gm-PBM.

Finally, we assessed microbial functional potential via heatmap-based predictive analysis. The COG heatmap (Figure 7H) showed that the AD group exhibited upregulation of functions related to “multidrug efflux pumps” and “cell wall-associated antibiotic resistance”, whereas both gm-PBM and tc-PBM interventions activated beneficial functional modules such as “ABC transporters” and “signal transduction components”, indicative of improved metabolic regulation. The KEGG pathway heatmap (Figure 7I) revealed that AD mice had suppressed activities in pathways such as “fatty acid biosynthesis” and “apoptosis”. Notably, gm-PBM treatment significantly restored key metabolic pathways including “secondary bile acid biosynthesis” and “glucose metabolism”, as indicated by red upregulation signals. These findings suggest that gm-PBM more effectively remodels gut microbial metabolic function, offering a mechanistic basis for its superior capacity to mitigate gut–brain axis-related inflammation.

### 3.8. Gm-PBM Reverses AD-Associated Metabolic Dysregulation via Metabolic Reprogramming

Encouraged by the finding that gm-PBM significantly restored microbial diversity and enriched SCFA-producing taxa in AD mice, we further conducted fecal metabolomics analysis across WT, AD, and gm-PBM groups. This was aimed at elucidating how microbial remodeling translates into potential metabolic mechanisms for ameliorating AD pathology.

**Figure 8:**
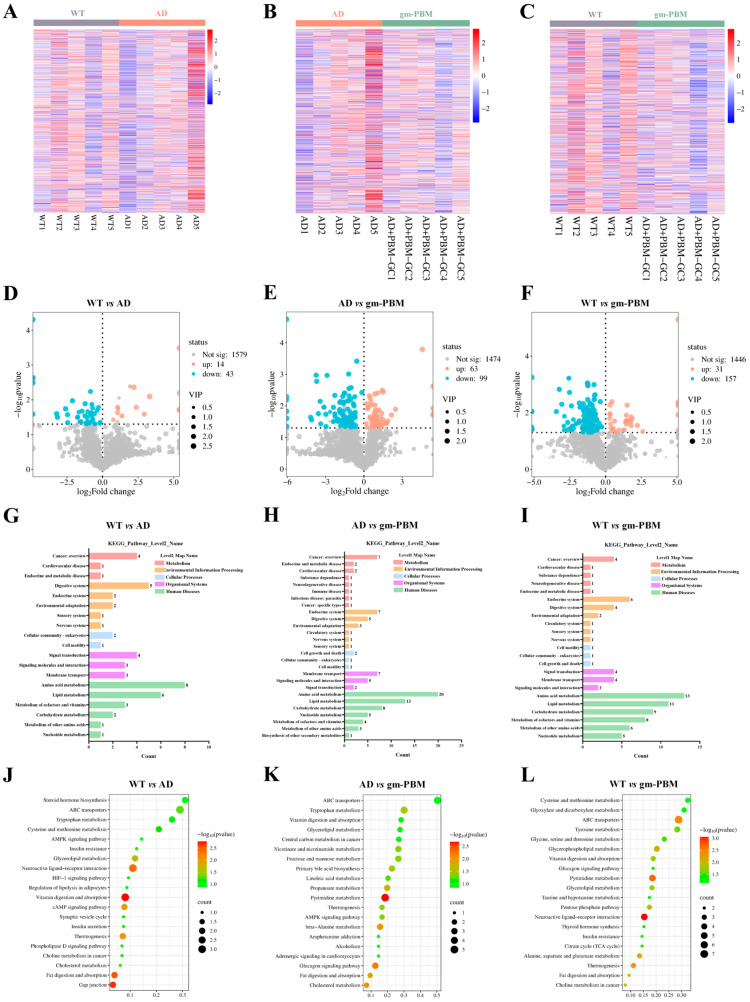
Gm-PBM intervention reveals potential mechanisms for ameliorating AD pathology through reshaping the fecal metabolome. (**A**–**C**) Heatmaps of differential fecal metabolites (Z-score scale; mean ± SD, n = 3 per group): (**A**) WT vs. AD, showing significantly upregulated (red) and downregulated (blue) metabolites between groups; (**B**) AD vs. gm-PBM, indicating metabolite level reversal after gm-PBM intervention; (**C**) WT vs. gm-PBM, demonstrating that gm-PBM treatment restores the metabolome profile toward WT-like patterns. (**D**–**F**) Volcano plots of differential metabolites (x-axis: log_2_ Fold Change; y-axis: −log_10_ *p* value; mean ± SD, n = 3 per group): (**D**) WT vs. AD: 14 metabolites significantly upregulated, 43 downregulated; (**E**) AD vs. gm-PBM: 63 metabolites upregulated, 99 downregulated; (**F**) WT vs. gm-PBM: 31 metabolites upregulated, 157 downregulated. Dashed lines represent thresholds of *p* = 0.05 (horizontal) and |log_2_FC| = 1 (vertical). (**G**–**I**) Bar plots showing KEGG Level 1 pathway classification of differential metabolites: (**G**) WT vs. AD; (**H**) AD vs. gm-PBM; (**I**) WT vs. gm-PBM. Bar height reflects the number of differential metabolites within each pathway. (**J**–**L**) KEGG pathway enrichment dot plots (dot size = number of differential metabolites in the pathway; color = −log_10_ *p*-value): (**J**) WT vs. AD; (**K**) AD vs. gm-PBM; (**L**) WT vs. gm-PBM. Metabolite screening criteria: *p* < 0.05, |log_2_FC| > 1. Statistical analysis: one-way ANOVA followed by Tukey’s post hoc multiple comparisons.

Compared to WT mice, the AD group exhibited widespread disturbances in the fecal metabolite profile (Figure 8A,D), with 14 metabolites significantly upregulated and 43 downregulated (*p* < 0.05, |log_2_FC| > 1). Following gm-PBM intervention (Figure 8B,E), 63 metabolites were significantly upregulated and 99 downregulated. Notably, in the WT vs. gm-PBM comparison (Figure 8C,F), 31 metabolites were restored to WT levels, while 157 showed inverse regulation relative to the AD group, indicating that gm-PBM effectively reversed AD-associated metabolic disruptions.

KEGG level 1 pathway classification (Figure 8G–I) revealed that AD mice showed marked downregulation in critical pathways including lipid metabolism, amino acid metabolism, and energy metabolism. gm-PBM intervention significantly reactivated pathways such as fatty acid biosynthesis, secondary metabolite biosynthesis, and signal transduction. Enrichment analysis (Figure 8J–L) further demonstrated that the WT vs. AD comparison showed significant enrichment in pathways related to MAPK signaling, tryptophan metabolism, and ABC transporters. After gm-PBM treatment, the AD vs. gm-PBM comparison revealed enrichment in fatty acid metabolism, PPAR signaling, and AMPK signaling pathways. Importantly, the pathway profile of WT vs. gm-PBM highly overlapped with that of WT vs. AD, suggesting a restoration of metabolic network organization toward a healthy state following gm-PBM treatment.

Collectively, gm-PBM markedly reversed fecal metabolic dysregulation in AD mice by bidirectionally correcting over 160 key metabolites involved in lipid, amino acid, and energy metabolism, and reactivating core metabolic pathways such as fatty acid biosynthesis and the PPAR/AMPK signaling axes. This metabolic reprogramming provides robust molecular support for the neuroprotective effects observed with improved gut microbiota diversity and highlights a potential mechanism by which gm-PBM alleviates AD pathology through a coordinated “microbiota–metabolite–signaling” axis.

## 4. Discussion

As a non-invasive method, light therapy is favored among a variety of physical interventions because of its non-contact and non-electromagnetic interference characteristics. Light therapies, including PBM and gamma frequency pulsed light intervention, have been suggested to improve the pathological damage of AD [12,15]. However, as a non-invasive treatment, the limited penetration depth of light into tissues especially via the skull is currently an obstacle to its clinical application [22,23]. We proposed that light regulation of the gut microbiota might indirectly treat AD through the gut–brain axis. The association between gut microbiota and AD has been widely recognized, and its underlying mechanisms have been increasingly elucidated through the gut–brain axis. Moreover, strategies aimed at modulating the gut microbiota—such as probiotics, prebiotics, and fecal microbiota transplantation—have shown therapeutic potential for AD [24,25]. Our previous work also demonstrated that PBM can ameliorate AD pathology by targeting the gut microbiota [20]. However, a critical question remains unanswered: whether gm-PBM, which exerts its effects indirectly via the gut–brain axis, can achieve therapeutic outcomes comparable to or even superior to those of tc-PBM, which directly targets the brain. To address this, the present study systematically compares the efficacy of gm-PBM and tc-PBM under identical light parameters and irradiation conditions.

Gm-PBM demonstrated significant effects in clearing Aβ plaques, increasing synaptic density, suppressing neuroinflammation, correcting gut microbiota dysbiosis, alleviating gut microbiota-associated inflammation, and enhancing intestinal barrier integrity. Its therapeutic efficacy was not only comparable to that of tc-PBM but also superior in terms of Aβ clearance within the hippocampal region. Previous studies have reported that visual stimulation and transcranial light intervention achieve Aβ clearance efficiencies of 40–60%, which is consistent with our findings: the Aβ plaque clearance efficiencies in the hippocampus were 67% for gm-PBM and 46% for tc-PBM [26].

Further 16S rRNA sequencing and metabolomics analyses revealed that PBM-GC selectively enriched SCFA-producing bacterial taxa, such as *Butyricicoccus*, *Lachnospiraceae NK4A136*, and *Eubacterium siraeum*, while significantly decreasing the relative abundance of pro-inflammatory genera like *Parabacteroides* and *Pseudomonas*. Correspondingly, there were notable increases in the levels of propionate, butyrate, and secondary bile acids. Functional prediction analyses further indicated that gm-PBM activated beneficial metabolic pathways including ABC transporter systems and AMPK-mediated thermogenesis, while suppressing inflammation-related modules such as multidrug efflux pumps and cell wall antibiotic resistance. Collectively, these microbiome and metabolic alterations suggest an indirect mechanism by which gc-PBM improves SCFA and bile acid metabolism, repairs intestinal barrier integrity, and subsequently lowers systemic inflammation. Such peripheral modulation is likely crucial in supporting the phenotypic transition of central microglia from pro-inflammatory (M1) toward anti-inflammatory (M2) states [27]. Interestingly, although tc-PBM effectively alleviated intestinal inflammation in AD mice, it failed to restore the eubiotic gut microbiota and even led to the transient enrichment of several conditionally pathogenic taxa. This may reflect a decoupling of immune modulation and microbial restructuring, suggesting that while tc-PBM can modulate host inflammation via neuroimmune pathways, it may not exert direct or selective effects on gut microbial composition. Future studies combining tc-PBM with microbiota-targeted interventions may help achieve more comprehensive gut–brain restoration [27,28].

Previous transcranial PBM studies predominantly emphasized central mechanisms involving mitochondrial cytochrome c oxidase (CCO) activation, gamma rhythm modulation, and microglial recruitment [29]. For the first time, our findings confirm that remote microbiome-metabolism modulation via the gut–brain axis can produce neuroprotective outcomes comparable to direct transcranial PBM, thereby significantly broadening the theoretical scope of PBM therapy. Furthermore, although our study employed continuous-wave irradiation, resulting in substantial cognitive and pathological improvements, additional research is needed to clarify the optimal therapeutic contexts and efficacy differences between various PBM waveforms (e.g., pulsed-wave versus continuous-wave modes) [30].

Nonetheless, our study has certain limitations. Firstly, although the APPswe/PS1dE9 mouse model is widely utilized in AD research, its Tau protein phosphorylation pathology is relatively mild. Additional validation using alternative models, such as 5 × FAD or 3 × Tg-AD mice, could further strengthen the generalizability of our conclusions. Secondly, although the use of 10-month-old WT mice was intended to match the cognitive status and gut microbiota profile of 6-month-old APPswe/PS1dE9 mice (given the accelerated disease progression in this model), we acknowledge that age may still act as an independent variable influencing behavioral outcomes and microbiome composition. Future studies should incorporate strict age-matching or longitudinal experimental designs to minimize this potential confounding factor. In addition, interventions such as fecal microbiota transplantation (FMT), targeted microbial modulation, or metabolite manipulation will be necessary to establish causal relationships along the gut microbiota–metabolism–immunity axis and to determine whether the therapeutic effects of PBM are mediated by microbiome alterations and downstream mechanisms [31]. Lastly, physical restraint required during light irradiation procedures in mice might induce stress responses, potentially affecting the accuracy of experimental outcomes. However, considering that such physical immobilization would not be necessary in clinical scenarios, we remain optimistic about the translational potential and clinical applicability of gm-PBM therapy for AD patients.

In addition, although gm-PBM induced measurable biological responses—including reduced Aβ burden, modulation of microglial activation, and partial restoration of gut–brain metabolic homeostasis—the cognitive benefits observed within the 4-week treatment window did not reach statistical significance compared with tc-PBM. This difference may be related to the disease stage and the indirect systemic mechanism of gm-PBM, which may require a longer therapeutic duration before translating into functional changes. Therefore, rather than suggesting inferior efficacy, our findings may indicate that tc-PBM and gm-PBM exert complementary and time-dependent effects: tc-PBM providing more immediate neural modulation, while gm-PBM may contribute to gradual systemic and neuroprotective benefits over time. Future studies with extended treatment periods or combinational PBM strategies may help clarify whether synergistic effects could yield more robust cognitive improvements.

## 5. Conclusions

In summary, this study is the first systematic comparison between gm-PBM and conventional tc-PBM in an AD mouse model. Our comprehensive analyses encompassed cognitive behaviors, cerebral pathology, gut microbiota metabolism, and immune-inflammatory responses, elucidating the multifaceted mechanisms underlying gm-PBM. The findings demonstrated that gm-PBM, without requiring skull penetration, achieved neuroprotective effects comparable or even superior to those of tc-PBM by modulating the gut microbiota–metabolism axis. Notably, gm-PBM exhibited pronounced advantages in restoring intestinal microbiome homeostasis and repairing gut barrier integrity.

This research expands the application scope of PBM technology in the field of AD treatment, providing essential theoretical insights and experimental evidence for developing a safe, noninvasive, cost-effective, and home-friendly intervention approach. Future studies will further clarify the core effectors, specific signaling pathways, and optimal dosage parameters of gm-PBM. Moreover, validations across multiple disease models and preclinical assessments will be undertaken to accelerate clinical translation. We believe that remote gut–brain axis-targeted phototherapy holds substantial promise for treating AD and other neurodegenerative disorders.

## Data Availability

The original contributions presented in this study are included in the article. Further inquiries can be directed to the corresponding authors.

## Abbreviations

The following abbreviations are used in this manuscript:

AD	Alzheimer’s disease
PBM	photobiomodulation therapy
PFC	prefrontal cortex
HIPP	hippocampus
APP/PS1	amyloid precursor protein/presenilin 1
LED	light-emitting diode
Aß	Amyloid beta
KEGG	Kyoto Encyclopedia of Genes and Genomes
